# Selections and Abstracts

**Published:** 1905-07

**Authors:** 


### SELECTIONS AND ABSTRACTS.

### Value of a Medical Library to the Community.*

By David I. Wolfstein, M.D.,
Cincinnati, Ohio.

There are catastrophes whose effects are eons deep and world-wide—genuine world sorrows. Such an one was the burning of the Alexandrian library.

As long as man shall bow in reverent homage at the shrine of knowledge, so long will he continue to mourn the loss of the priceless treasures of Alexandria. What precious records of the older civilizations were thereby destroyed no one will ever know; how much the progress of humanity was thereby blocked we can hardly even surmise. In no way, however, are we made more conscious of our dependence upon libraries than by recalling the grief still poignant—yes, infinitely keener now, after sixteen centuries, than in the days when this crime against society was still moderately young.

The word *library* can not be thought or uttered without awakening its associated concepts of culture, of progression, of achievement; for who will not agree with Bacon "that libraries are the shrines where all the relics of the ancient saints, full of true virtue and that without delusion or imposture, are preserved and reposed?"

With magic touch the skilled painter limns in his perspective, deceiving our senses with the illusion of vast distances; in the perspective of knowledge there is no deception. In countless tomes on groaning shelves the human mind has recorded for our benefit the mute, though eloquent evidences of its incessant activity. Sometimes from written manuscript or printed page there flashes forth the words of some great genius, serving by a brilliancy which never pales to perennially illuminate the dark abode of ignorance; or, again, it may be only the less luminous inscriptions of some

* Read before the Academy of Medicine of Cincinnati, April 3, 1905.

ancient plodder adding perhaps only some isolated fact to stimulate, as time rolls on, some greater mind to greater achievement.

The *historical perspective of human wisdom* is furnished by books, and books mean libraries.

The theme assigned me for this evening is the value of a medical library to a community, and my only reason for responding to it was the hope that the little I shall say this evening might serve to awaken an increased interest in our own excellent institution.

It is certainly superfluous that any one should appear before this audience in advocacy of such a self-evident proposition. Whilst, however, the profession here and elsewhere believes in the truth of this proposition abstractedly, it is very doubtful if the actual use made of our library justifies the assumption of any other than a very luke-warm belief.

Unquestionably, the exigencies of modern social existence force each medical individual to develop primarily his earning power, and probably everywhere, but especially in this country, the tendency is growing to render unto the Cæsar of science the things which are Cæsar's, whilst the practitioner is satisfied to be that which the name implies. If so, it is an evil tendency, and will be of short duration. The giants in medicine and the real leaders in every community have, as a rule, always successfully applied theory to practice; indeed, in only too many instances the theory of some great practicing clinician has been far in advance of the corroborative scientific demonstration of its truth.

There is no antithetic relationship between theory and practice, and the slave of the lamp has usually been the master at the bedside.

Virchow has said: "All scientific research is really literary." It is the settled though somewhat absurd custom of society to measure the scientific achievement of each epoch by the names of its few pre-eminent leaders. We are rendered conscious of the existence of that subtle, universal, mysterious force—electricity—by the sudden flash of the lightning, but what immeasurable volumes of power surged restlessly between opposing poles before the jagged stroke was born.

As the lightning flash is but the resplendent culmination of this great potential force, so, too, comes the genius, hero of his epoch, revealer of new truths, and the world, all forgetful of the vast stores of knowledge garnered through all the ages, laboriously accumulated by toiling dwarfs in many fields, has encomiums for him only whose greater stature lifts him above the mass of humbler workers.

The present generation may be likened unto a child in a crowd raised upon the shoulders of the elders that it may command the better view. "Grant them but dwarfs, yet stand they on giants' shoulders and may see the farther." So also must the science of to-day stand upon the shoulders of past achievement, and whilst with confident hope it looks ever to the future, it must oft perforce turn its wondering and worshiping gaze backwards, even to the very birth-time of the race. The spirit of the living present, mindful of the magnitude of its material triumphs, with the blood of jubilant youth in its veins, is often prone to hold in light esteem the labors of past centuries. Our greatest geniuses and our most original men in science have, however, cheerfully acknowledged their dependence upon the achievements of their predecessors.

To the uninitiated cellular pathology spells Rudolph Virchow, but this cornerstone of modern pathology rested securely upon the foundation laid down by Schwann and Schleiden. I have had no greater enjoyment than reading in many of Virchow's addresses his masterly account of the evolution of pathology and the exact statement of our indebtedness to the pioneers in this branch.

Listen to Virchow's own words: "The reproach has often been made that I have too much endeavored to make modern views conform to the older theories, but I can say here with a clear conscience that I have as little desire to rehabilitate Galen or Paracelsus as I should refrain from openly acknowledging that which is true in their own views and experiences."

For the legion of the partially instructed the evolution theory begins with Darwin, whereas Darwin, like Virchow, was conscious of the rich heritage of knowledge bequeathed him by the past. Those who have followed the sequence of scientific events which led up to the *great theory*, worship Darwin none the less because he was the gifted architect that builded out of the vast material collected for him by many minds of lesser originality that impregnable fortress behind whose solid bastions the eternal verities of science now fear no attack. But before Darwin there were Linneus, Cuvier, Agassiz, Gœthe, Oken, Lamarck and Lyell.

And so before Pasteur there were Spallanzani and Ferdinand Cohn. Before Hitzig illuminated the obscure domain of cerebral physiology by his splendid experiments he was master of all the pre-existent recorded experience and experiment. Through Bouillaud, in 1825, he knew that destruction of a small excentric part of the brain might cause the symptom-complex of aphasia. He had profited by the epochal observations of Broca in 1862, and beyond all he had been impressed with the keen deductions of Hughlings Jackson.

This is what Hitzig himself says of Jackson: "As for Dr. Jackson, there is no doubt that his merits are of the most manifold character, but in foreign countries we connect his name above all with the memory of one fact, namely, that he was the first one who referred the partial cortical convulsions to peculiar centres of innervation. Many another had already observed these convulsions, but he alone hit the right explanation for the phenomenon, and by referring the convulsions to such centres he argued the existence of motor centres in the brain, and even told to a certain degree about what their condition was, before physiological research was able with certainty to prove what they were. When this society summoned me to be the first of these lecturers it doubtlsss did so for no other reason than because I was precisely the first one, now some thirty or more years ago, to confirm by physiological experiment and to define more closely that which Hughlings Jackson had already concluded from clinical facts."

The prime function of the library is, therefore, to conserve the wisdom of the past for the use of the present. Osler says: "It is hard for me to speak of the value of libraries in terms which would not seem exaggerated. Books have been my delight these thirty years, and from them I have received incalculable benefits. To study the phenomena ot disease without books is to sail the uncharted sea, while to study books without patients is not to go to sea at all. To be of real value, there must be a general and continuous use; the books must be made available and their use attractive. What exsuccous attenuated offsprings books would be but for the pabulum furnished through the placental circulation of a library!"

The medical student should be inoculated during his college career with the love of books, authoritative books, and as a practitioner he should, wherever possible, maintain and increase his affection for our medical classics.

If we have ever analyzed the motives that impelled the majority of us to choose medicine as our vocation, what were they? It was surely not the hope of great financial reward. I should say that any young man who would with deliberation select medicine as a field in which to pile up wealth, would, judged mildly, be open to suspicion of intellectual immaturity, and judged properly I should say he was already presenting a symptom of dementia precox, the psychosis of the juvenile and adolescent periods.

There is also a well-recognized but debatable emotional state known as the "inward call," supposedly operative in recruiting the ranks of the clergy, but I doubt its influence in bringing accessions to our own profession. The laity loves to think so, as, indeed, they attribute generally a sum of altruistic motives to us I fear quite beyond our real deserts. Then, again, another small percentage drifts into medicine as the final culmination of that mental condition of delightful indecision which actuated the conduct of the celebrated Micawber.

Still another group adopts medicine because of an inherent distaste for commercial life and because it is a gentleman's profession. What governs the great majority of us, however, though as an imperative concept it may not have received full recognition as a dominant idea, is real sympathy with the love of study. Even in the humbler contingent of novitiates there is a certain degree of affinity for the intellectual side of life. Medicine is attractive, because, whilst it fosters distinctly the intellectual, there is a fair guarantee of reasonable practical success coupled with a life of study, and eminent respectability. In every community, rural or urban, there is always some young man who is fond of study, loves books, is mayhaps of speculative mental habit, and such an one in determining his future avocation will often turn to medicine. Especially is this true in the country and smaller towns. Why, then, if the love of study and desire of knowledge be the magnetic power that attracts at the outset of a career, should we see these noble tendencies languish and wither in later years?

Why is the *Wissenshunger*—hunger for knowledge—of youth succeeded by the intellectual anorexia of a later period? It is largely because, except for a favored few, blessed of the gods, whom Fortune has placed in a proper intellectual environment, their hunger for knowledge is fed from the very start with the dry husks of learning, because they are forced to subsist upon a mind-food which produces first mental neurasthenia, psychasthenia, and, except in very resistant brains, often ends in premature mental sterility. First, the interminable cramming of the ordinary medical school, then the special "stunts" for the examinations, are responsible for much of the mental inertia which follows.

If this state of mental polition be once established, then the intellectual provender of the *average* physician is the innutritious material of the *average* journal. I leave it to the honesty of most of you to indicate to yourselves the real value of this class of reading. The valuable reading, the reading that informs, that broadens the intellectual horizon, that creates that glorious feeling of mental plenitude—"of good measure, pressed down and running over"—is of another character.

Who would wish to command his subject, reads on subjects, not with superficial diversity, spreading himself out thin over too wide a field, but with concentrated energy along single lines.

In cultivating for the knowledge crop intensive farming pays better than diversified farming. A reader of this school will treasure our great medical classics, will study the contributions of the authors in the original. If he wishes first a broad view, a general acquaintance with a subject, he will consult such magnificent encyclopedias as Eulenburg or Ziemssen's, or our own "Reference Handbook," or others of like scope.

Of greater value still are the many special monographs. For reading of this character and students of such calibre, a medical library is indispensable.

One of the most satisfactory signs of the medical renascence in this country—and I use the word renascence advisedly, for I verily believe that medical scholarship, and especially the literary standard, were higher in America at the beginning of the nineteenth century than in the middle of it—is the wonderful growth all over our land in medical libraries. There are now about one hundred and twenty medical libraries in the United States, some of which started in a small way, and, fostered with enthusiasm and loving care, have reached splendid proportions.

The Boston Medical Library, the Brooklyn Library, the Library of the New York Academy of Medicine, and others in Chicago, Cleveland, Baltimore, Indianapolis, Grand Rapids and Denver, are fine examples.

These excellent book collections have all grown up, out of, and around their respective societies, and represent the object of their special pride. For the proper utilization of medical literature the first essential is a complete series of the best archives and journals. Every day such complete series are becoming rarer and more expensive. Only a library with a good income can hope to maintain even the absolutely indispensable periodicals.

Text-books are of ephemeral nature. A few, such as Foster's "Physiology," or Osler's "Practice," or Gower's "Nervous Diseases," and, of course, others of like fame, will always be more or less standard.

The subject-matter of most text-books, however, is, as a rule, fairly *passè* when it appears. To keep text-books up to date frequent editions are called for, which is only possible for the most popular.

In the great journals and archives and monographs, on the other hand, there is a live literature. This is the forum of first debate. Herein the newer views and great thoughts are forged in the white heat of creative fire. In these contributions the author displays his methods, shows us his process of reasoning, and step by step we may follow his researches or his logic, and at every step our own powers of criticism are awakened.

It has been estimated that nine-tenths of the ordinary journalistic productions is practically worthless a few years after it has been written. I am confident that five-tenths is certainly worthless the day it appears. Nevertheless, the chaff must be preserved with the wheat, and it is only in a well-conducted library that a moderately complete collection can be maintained.

A library will be of value to the medical community when it keeps all the best reference works on the bibliography itself. In this, as we all know, the United States has taken first degree. The Index Medicus, and the library of the Surgeon-General's office are the paradigms in this line. Our Hospital Library has them, and besides, such tried and true friends as *Schmidt's Jahrbuck*, *Virchow and Hirsch Jahresbericht*, *Jarbuck der Gesammten Medicin*, *Archives Generale de Medicine*, etc.

One form of medical literature in which we are especially deficient, and which only a few private individual medical Crœsuses can possess, and which I regard as the most valuable of all types, are the transactions of the great general and special societies. In my own branch of neurology the very best productions, those that are epoch-making, appear in these "Transactions," and even the best journals rarely have more than inadequate reviews. My respect for the review is not profound. I review myself. No one can do your work for you as well as yourself. True, they point the road to the good literature, but how few of us ever make the journey!

If you would gain inspiration go to the original source; drink deep of the spring of knowledge as it sparkles forth clear and limpid, not after it has become contaminated and stagnant by much wandering.

To make a library truly serviceable there must be in charge a librarian who can serve. *He must be something more than a book shepherd.* True, he must love his flock of books, as books, but more for the meat that is in them than for the hide in which they are bound. A librarian should have intellect enough to serve us as an intelligent guide and know his material well enough to place it promptly at our disposal—yes, even to suggest that for which we are searching. Personally I wish that we could endow a librarian with plenary powers. He should, of course, have *constructive power*, for his chiefest aim should be to build up his library. Within wise limits, however, "and there's the rub," he might be endowed with *destructive power*.

We are all familiar with those doctrinaires who in their goodness of heart wish to benefit humanity by slaughtering the unfit. Unquestionably, it would be vastly beneficial if the idiotic, the hopelessly degenerate, the hardened criminals, and all that great horde of extra- and anti-social unfortunates who are the millstones around the neck of society could be exterminated.

Although this school of superstrenuous Spartans has generously offered to allow the medical profession to act as executioners, we have not yet availed ourselves of the privilege. We have felt that there might be some practical difficulties to overcome. Huxley say, in "Evolution and Ethics:" "I have been obliged to admit this that rigorously scientific method of applying the principle of evolution to human society hardly comes within the region of practical politics, not for want of good will on the part of a great many people, but because, for one reason, there is no hope that mere human beings will ever possess enough intelligence to select the fittest. The 'points' of a good or a bad citizen are really far harder to discern than those of a puppy or of a Shorthorn calf."

Whilst I cheerfully relinquish the hope that society will ever adopt this drastic plan of the Spartans for its perfection, the librarian would be less hampered with practical considerations in connection with the preservation of *his* fit and unfit types. Think what blessing would be conferred if a well-balanced librarian or a congenial committee with incendiary inclinations could hold an annual *auto da fe* and burn publicly in the market-place all the hopelessly degenerate, unfit and useless matter which contrariwise must encumber the shelves of the library and perhaps—awful thought—propagate a numerous progeny in kind!

The word Academy took its origin from a certain garden belonging to the Greek Academos, "See there fhe olive-groves of Academos, Plato's retreat." Here, surrounded by all that was helpful, beautiful and inspiring, Plato expounded his philosophy. The groves of Academos became the very center of Greek wisdom.

So, too, our Academy should be the center of all that is fruitful and useful for the medical profession of this city. I must refrain from encroaching upon the territory of the next reader, but I trust the time is near at hand when this Academy may stake out for itself larger duties and more important aims.

The medical library of this city should be under our control. We ought to have—if not our own building, and that is hardly feasible—quarters of such fitness as would furnish a safe repository for the splendid collection of books already existent here.

The profession here should foster not only the scientific, but the social side of our lives. Our Academy should be at once our school and our club. When we meet to hear our papers read the friendly, honest faces of our literary companions should greet us from loaded shelves around the room, whilst from the walls the honored countenances of our great medical heroes should inspire us to noble emulation. The rooms of the Academy should be open day and night, and as few restrictions upon the use of literature as is consistent with the maintenance of the library should be established.

There should be cozy, quiet corners, comfortable chairs and fine tables, and at intervals, instead of being devoted solely to library uses, they should be ranged round the festal board. I know that physicians have a hard struggle and not much money to spend, but who would not pay higher dues for the privilege for such an Academy?

In other cities they maintain extra library dues, with a different scale of cost for active, for non-resident, and for associate library members. If we had a fireproof library or quarters in a safe building, and it was generally known that the library was the child of the Academy, we should soon be deluged with donations.

Duplicates would accumulate, which we could exchange for missing files with other libraries, and I have no doubt that the Public Library would, as soon as it felt safe in so doing, abandon to us its medical department, and the requisite funds for its support through taxation might be allotted to us, and would, of course, go far towards the support of our own institution. The details could be probably worked out, and there is no reason to doubt that we should have the same success in Cincinnati as has been obtained in other cities, amongst them cities of lesser importance than our own.

Gentlemen, the medical organism of Cincinnati has not been as flourishing as the high standard of professional ability and the importance of this commonwealth would justify, but it would almost seem as if a healthier condition might be predicted in the near future. In the meantime, whilst we are hoping—I might even add praying—that the great leaders in this community may see their way clear to the establishment of medical affiliation and co-operation of all the forces that are here existent, there is one clear duty for this Academy.

We must erect a home, and in that home the features of greatest attraction must be the library. The influence which would radiate from such an Academy, installed in its own building and surrounded by ideal conditions which every member must feel to be the noblest part of our lives, could not fail to arouse renewed interest along other lines of medical activities.

On this burning question, at least, the Academy can act unitedly, and it would be but a few years before the whole profession would receive inestimable benefits and could bequeath to those who follow in our footsteps a useful and noble institution of learning and advancement.—*Lancet Clinic.*

---

### A WARNING AND A PROTECTION FOR X-RAY WORKERS.

A. Holding (*Medical Record,* March 26, 1905), rehearses the dangers of dermatitis, malignant disease, azoospermia, etc., that confronts the X-ray worker, and points out that it is possible that the second decade of experience with Roentgen's discovery may reveal activities as yet unsuspected. The greatest care should be observed by radiographers to avoid unnecessary exposure, and the author describes and illustrates a suitable screen, three feet by six in size, covered with double layers of lead pipes, which is intended to cut off all rays from the operator who manipulates the switchboard from its shelter and observes patient and tube by means of a pivoted mirror affixed to the side of the framework.

M. A. B.

### BROMIDE POISONING.*

BY C. C. STOCKARD, M. D., ATLANTA, GA.

During the past few years I have seen a dozen or more cases of bromide poisoning, some of them resulting from a lack of knowledge on the part of the physicians of the physiological effects and toxicology of the drug, and others showing that the laity look upon bromides as harmless remedies.

The number of cases seen by me, together with the failure on the part of physicians to recognize the condition, and the real seriousness of it, have led me to the conclusion that a paper calling the attention of the profession to the subject and pointing out the symptoms by which bromidism may be readily recognized would be of real value.

Some of the cases have resulted from the use of bromides in efforts to get release from the morphine habit. About six years ago there was reported a case in which by some sort of mistake a woman took three ounces, or more, of sodium bromide within about forty-eight hours. A deep sleep of four or five days' duration followed; and, after coming out of this, it was found that she did not feel the need of morphine, to the use of which she had long been addicted. As a result of reporting the case a good many have been treated in this way; and, I doubt not, a number of deaths have resulted. But, outside of these cases, there seem to be a good many occurring in the practice of physicians either from idiosyncrasy of the patient or from taking the drug too freely when the physician has given directions in a rather loose way; and, still others have taken it of their own accord, thinking there is no harm in bromides.

The cases coming to me from other physicians were not at all recognized by them as cases of bromide poisoning. In fact, the bromide was generally being pushed, to control the symptoms thought to be delirium tremens, or some other like condition. When bromide is taken in sufficient quantity, within two or three days' time, to produce sleep lasting several days, the patient first

*Read at the annual meeting of the Medical Association of Georgia, at Atlanta, April 19 to 21, 1905.

becomes drowsy, tremulous and weak, frequently dropping things from the hands, and walking with an unsteady gait and dragging of the feet. The talk is drawling, the tongue seeming to be thick and inactive, the eyes droop, and the lids are generally gummed. This condition is followed by a deep sleep, lasting from a day or two, to longer, according to the amount of the drug taken. In one case, in which the patient took the drug several days, the last dose being nearly an ounce, a profound sleep continued for two weeks.

During the sleep the patient lies usually very quiet, rarely turning even. The breathing and pulse are regular, slow, and full. The bladder and bowels are emptied involuntarily, and without at all arousing the patient. The breath has a peculiarly foul and metallic odor, which is usually so characteristic of the condition that it may be at once recognized by this odor. The tongue is covered with a heavy, white, slimy coating, which gives way later to a red, glazed condition; this not occurring, however, for a week, or it may be, two; and by the time it does occur, the peculiar odor of breath has disappeared. Coming out of the sleep, there is the same slow talking, with dragging of the limbs and letting fall of articles from the hands, as described above, and with it hallucinations, usually of persecution. The patient imagines he is being hunted, and is going to be killed, or something of the kind. He weeps, saying he has done no one harm; and, though he seems harmless, at times he is ready to fight and, unless properly guarded, is liable to do harm. This state resembles very much what is frequently seen as the result of alcoholism—that is, the mental condition—but the breath, the thick tongue, and the dragging voice and walking readily distinguish the two.

When bromide is continued sometime in rather large doses, but smaller than above described, the condition that follows the sleep is reached without going through it, the patient simply sleeping more than usual and developing a foul tongue, with the breath, muscular weakness, etc., above described, together with the mental disturbances. I have seen several cases of this kind occurring in patients for whom bromide had been prescribed with same latitude as to interval between doses. With one exception, all the cases I

have seen recovered, in from a day or two to three weeks; but one case—which had been heavily dosed for several weeks and had developed the characteristic symptoms, and was dosed still more freely while being brought to the city, in order to keep him quiet—went, within a few hours after entering the sanatorium, into a coma, and died within less than forty-eight hours.

While to call attention to the seriousness of the too free use of bromides is the prime object of this paper, it may be well to say that a knowledge of the effect will enable the physician often to produce a most desirable sedation, just coming short of serious toxic effect. To accomplish this, about 1½ to 2 ounces should be given within, say, seventy-two hours. Taken thus, the effect will show itself but slightly before the entire amount has been given, or, possibly, even the next day. In persons who are sea- or car-sick, there is nothing known to me which approaches the bromides in efficacy, and there are other conditions in which to produce such a decided sedation of several days' duration would be of immense benefit. I think the above amounts can be safely given, and I have never seen the mental condition upset follow this dosing, but it is about as far as one can go with impunity.—*Merck's Archives.*

---

### HAMAMELIS—ITS THERAPEUTIC USES AND THE POISON PERILS OF ITS ADULTERATION.

In an address delivered April 26, 1905, before the Danbury Medical Society on "The Practical Value of Old Remedies," John V. Shoemaker, M.D., LL.D., of the Medico-Chirurgical College, Philadelphia, Pa., spoke of witch-hazel, or hamamelis, in the following terms:

"Witch-hazel, or Hamamelis Virginiana, an excellent old-time remedy, has a well-defined range of usefulness within which it is without a rival. Externally and internally it is sedative and astringent. It is used as lotion and ointment in many diseases and injuries of the skin, in leg-ulcer and varicose veins. It is serviceable in acute and chronic diarrhœa, internal hemorrhages, bronchorrhea, epistaxis, varicose veins and varicocele.

"The distilled extract of hamamelis is a valuable application to sprains and bruises. Hamamelis is very useful in checking epistaxis, bleeding sockets after the extraction of teeth, bleeding homorrhoids and many other forms of hemorrhage. An ointment containing witch-hazel is of service in burns, eczema, erysipelas, sunburn, seborrhea, acne, etc. A diluted fluid extract of hamamelis makes an effective lotion in hyperidrosis. A witch-hazel lotion or ointment is an excellent application in fissure of the anus. When given internally this remedy exerts the same astringent and sedative action, and is highly valued in the treatment of acute and chronic diarrhea, dysentery, hemorrhage from internal organs, purpura hemorrhagica, varicose veins and ulcers and varicocele."

In his standard work on "Therapeutics," Hare also states that it is "a plant of extraordinary remedial power," and adds the following highly significant declaration:

"The one official preparation of the U. S. P. is the fluid extract (*Extractum Hamamelis Fluidum, U. S.; Liquidum, B. P.*), dose 5 to 20 drops (0.35-1.30). The dose of the distilled extract, which is not official and is a perfectly clear liquid, is from 30 drops to 1 drachm (2.0-4.0), and this is much the best preparation for internal and external use. *Unfortunately, the preparations of the drug vary very much in both odor and efficacy. Some of the proprietary preparations of witch-hazel are more active than those ordinarily dispensed in the drug-store. This is due to greater care in their preparation and to the fact that they are sold in original packages without exposure to the air.*"

The truth of Hare's contention that the common commercial witch-hazels of the retail drug-store vary as to both odor and efficacy, and, for the reasons given, are less active than the proprietary article, as well as the peril of the common commercial and unidentified witch-hazels of the market, has been emphasized in startling fashion by such well-known and creditable medical investigators and expert witnesses as Buller and Wood, whose voluminous report to the American Medical Association on the adulteration of witch-hazel and other medicinal extracts, etc., by wood alcohol and formaldehyde, has attracted the attention of the medical profession and press throughout the country, and still continues to excite the interest and evoke the comment of both.

As an example of the extent to which this adulteration has been carried by unscrupulous manufacturers of the common commercial and unidentified varieties of the product, it may be stated that of seventy (70) samples recently purchased from as many wholesale and retail dealers in the commercial article in six of the largest cities in the United States, scrupulous investigation by the most reputable analytical chemists of the country showed that fifty-two (52) of the samples in question contained wood alcohol (poison) or formaldehyde (poison), or both.

In discussiug this question, the "*Bulletin of Pharmacy*" expresses the opinion that "the practice of substituting wood alcohol for grain alcohol in the manufacture of medicinal preparations is a most insidious and pernicious means of poisoning, and the sales of remedies and toilet preparations manufactured or adulterated with wood alcohol should be restricted by law."

Until, however, such laws be enacted by all States, it should be obvious, in the face of the pernicious conditions described, that the perils of the poisons specified—as is suggested by Hare—may be avoided in cases in which Hamamelis is indicated only by prescribing exclusively a standardized proprietary product of guaranteed and invariable purity, quality and strength such as Pond's Extract of Hamamelis, which embodies and possesses all the qualities and advantages indicated by Hare as accruing from the greater care exercised in its preparation and the fact that it is sold only in original packages, without exposure to the air.

Pond's Extract of *Hamamelis Virginiana* (as the plant is botanically described by Hare and Shoemaker, or *Virginica*, according to Coston) has been relieving pain and performing other beneficent functions in the conditions indicated by Shoemaker for the past sixty years. While it has been imitated and substituted in every conceivable form during this extended period, it stands out to-day all the more efficient and esteemed by such comparison, and, in addition to its superlative medicinal properties and action, is a positive guarantee to physician and patient alike against any and all of the poison perils of the common commercial witch-hazels so vividly portrayed by Buller, Wood, Darlington, Lloyd, Hare, Gamble, *London Lancet*, *Journal of the American Medical Association*, *Medical News*, *Medical Record*, *Therapeutic Gazette*, *Boston Medical and Surgical Journal*, *Druggists' Circular*, *Bulletin of Pharmacy*, *Western Druggist*, and numerous other medical writers and professional publications of equal standing and authority.

